# Retrospective review of spinal magnetic resonance images to determine the margin of safety for epidural analgesia in pediatric patients

**DOI:** 10.1016/j.bjane.2025.844687

**Published:** 2025-10-17

**Authors:** Noah Letofsky, Dana Archibald, Anthony M.-H. Ho, Lais Helena N. e Lima, Rodrigo M. e Lima, Vinicius C. Quintão, Fernando B. Cançado, Ricardo V. Carlos, Leopoldo M. da Silva, Fernando N. Bellicieri, Saullo Q. Silveira, Arvin Haghighat, Rachel Phelan, Glenio B. Mizubuti

**Affiliations:** a Queen’s University, Department of Anesthesiology and Perioperative Medicine, Kingston, ON, Canada; b University of Manitoba, Department of Anesthesiology, Perioperative, and Pain Medicine, Winnipeg, MB, Canada; c Universidade Estadual Paulista (UNESP), Faculdade de Medicina de Botucatu, Departamento de Anestesia e Especialidades Cirúrgicas, Botucatu, SP, Brazil; d Universidade de São Paulo, Faculdade de Medicina, Disciplina de Anestesiologia, São Paulo, SP, Brazil; e Universidade de São Paulo (HC-FMUSP), Faculdade de Medicina, Hospital das Clínicas, Instituto da Criança e do Adolescente, São Paulo, SP, Brazil; f Hospital São Luiz – ITAIM / Rede D’Or – Equipe de Anestesia CMA, Departamento de Anestesiologia, São Paulo, SP, Brazil; g Hospital São Luiz – Jabaquara / Rede D’Or – Equipe de Anestesia CMA, Departamento de Anestesiologia, São Paulo, SP, Brazil; h Queen’s University, Department of Diagnostic Radiology, Kingston, ON, Canada

**Keywords:** Epidural analgesia, Magnetic resonance imaging, Patient safety, Pediatrics, Retrospective study

## Abstract

**Background:**

Deeply sedated children cannot provide feedback if an epidural needle traumatizes the Spinal Cord (SC). Knowing relevant structure depths may, therefore, improve safety. We aimed to determine the epidural margin of safety, i.e., distances from the Ligamentum Flavum (LF) and from the dura mater to the SC in pediatric patients measured (i) Perpendicular to the SC and (ii) Parallel to the spinous process (to approximate needle trajectory).

**Methods:**

Retrospective review of pediatric (0‒12 years-old) T2-weighted sagittal MRI spine scans without spinal pathology. Three investigators independently measured distances from the ventral edge of the LF, and from the ventral edge of the dura mater to the SC at T5/T6, T9/T10, and L1/L2. All measurements were taken perpendicular to the SC and parallel to the angle of the spinous process of the inferior vertebra.

**Results:**

111 MRI scans [52 females, 0.08‒12 (median 7) years-old] were analyzed. The conus medullaris was identified superior to the L1 vertebra in 47 scans, requiring L1/L2 measurement exclusion. When all ages were combined, the largest median (range) depth [dura-mater–SC = 4.87 (2.30‒10.30) mm, LF–SC = 8.10 (4.57‒12.53) mm, measured perpendicular to the SC; and dura-mater–SC = 8.20 (3.75‒19.57) mm; LF–SC = 13.40 (5.50‒39.77) mm, measured at the angle parallel to the inferior spinous process] was at T5/T6.

**Conclusion:**

Our results suggest that the margin of safety (dura-mater–SC distance and LF–SC distance) for performing epidurals in children may be greatest at the mid-thoracic spinal region. The measured ranges were very wide. Further studies are warranted to validate these findings in pediatric patients with other relevant “epidural placement” positions.

## Introduction

Perioperative epidural analgesia is an important option for pediatric patients.^1^, ^2^, ^3^ Despite very low rates of undetected/inadvertent catheterization of the subarachnoid space and needle/catheter trauma to the Spinal Cord (SC), the potentially catastrophic consequences of such occurrences warrant attention.^3^ The challenges of pediatric epidural placement include patient size, smaller distances between critical anatomic structures, increased compliance of the rib cage, reduced rigidity of the Ligamentum Flavum (LF), and asleep insertion with lack of patient feedback.^4^

It has been suggested that knowledge of the distances from the skin to the dura mater (or simply, “dura”) and from the dura to the SC may improve safety while performing epidural analgesia.^5^ Four studies have examined this topic in adults^6^, ^7^, ^8^, ^9^ and three in children.^5^^,^^10^^,^^11^ These studies have reported distances from the skin to the epidural space and from the dura to the SC. However, to our knowledge, no studies have examined the distance from dura to SC in children or the distance from LF to SC in adults or children, both of which we believe are relevant to the safe placement of all epidurals. Additionally, previous pediatric studies^5^^,^^10^^,^^11^ limited the measurements of thoracic spine anatomy to ≤ 8 years of age. To address these knowledge gaps, and recognizing that children > 8 years-old also undergo major truncal surgeries that could benefit from epidural analgesia, we aimed to determine the margins of safety for neuraxial anesthesia techniques in pediatric patients aged 0‒12 years by measuring the distances at thoracic (T5/T6 and T9/T10) and lumbar (L1/L2) levels on Magnetic Resonance Image (MRI) scans from (i) The ventral edge of the LF to the dorsal edge of the SC, and from (ii) The ventral edge of the dura to the dorsal edge of the SC. T5/T6 is an appropriate level for thoracotomies and chest tubes and may also encompass chest trauma-related rib fractures, while T9/T10 and L1/L2 are suitable levels for upper and lower abdominal surgeries as well as threading the epidural catheter to higher (thoracic) levels. In previous adult studies, the dura-SC distances were found to vary widely. For instance, at T6/T7, the range in 19 patients (supine position) was 2.1‒7.5 mm^12^ and the range in nine volunteers (supine position) was 4.3‒15.8 mm.^9^ Similarly, we hypothesized that wide distance ranges would be found at certain spinal levels in children. As the study was not comparative, no power analysis was performed and only a convenience sample was used.

## Materials and methods

**Figure 1:**
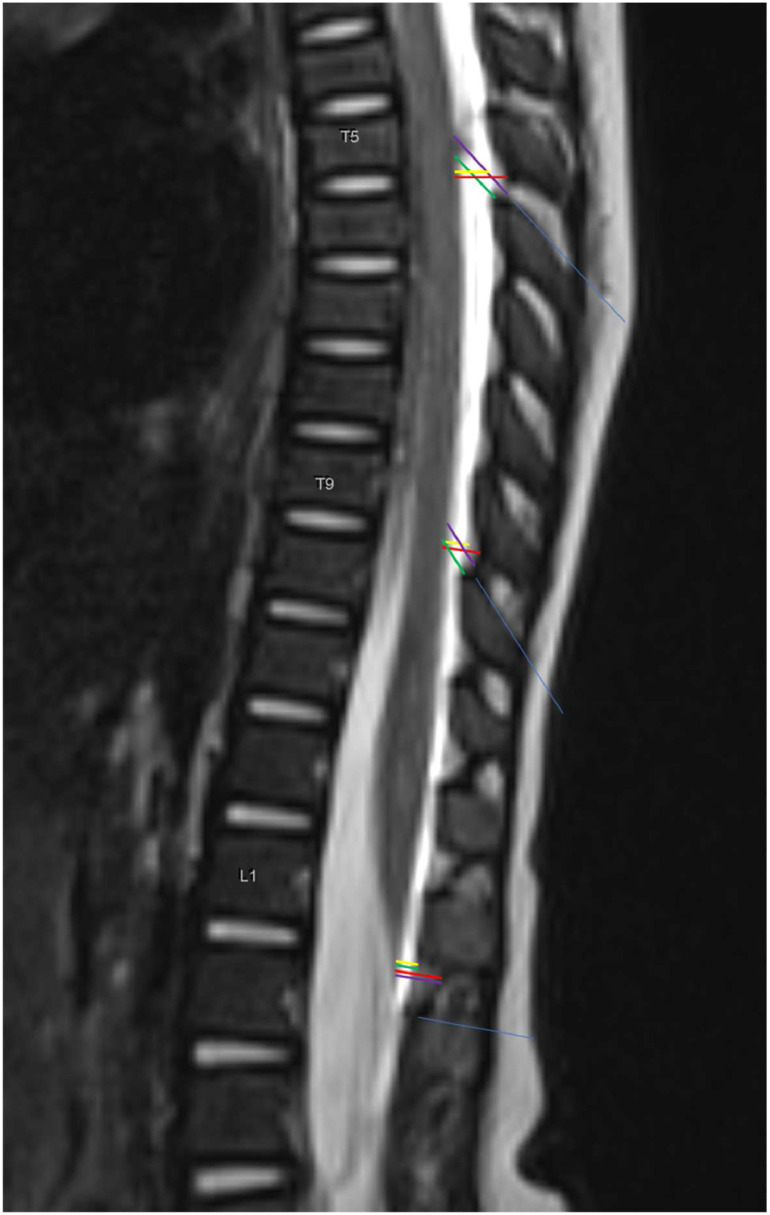
T2-weighted sagittal MRI spine image demonstrating 4 measurements/distances measured at levels T5/T6, T9/T10, and L1/L2: (1) dura-SC perpendicular to the SC (yellow line); (2) LF-SC perpendicular to the SC (red line); (3) dura-SC parallel to spinous process (green line); and (4) LF-dura parallel to spinous process (purple line). The long/blue line represents the line parallel to the inferior spinous process used as reference for measurements (3) and (4). Dura, Dura mater; SC, Spinal Cord; LF, Ligamentum Flavum.

Following institutional research ethics board approval (Queen’s University Research Ethics Board protocol 6029558, Hospital das Clínicas HCFMUSP CAAE 65178522.1.0000.0068, Rede D’Or CAAE 83100824.5.0000.0087), we retrospectively reviewed T2-weighted sagittal MRI spine images from 111 patients (0‒12 years-old) from three institutions (Kingston Health Sciences Centre, Kingston, Canada; São Luiz Hospital, São Paulo, Brazil; Hospital das Clínicas HCFMUSP, São Paulo, Brazil) who had suspected spine pathologies but whose MRI scans were within normal limits. The convenience-based sample size consisted of all eligible patients undergoing MRI scans between January/2013 and January/2023. No statistical power calculation was performed prior to data collection. Images were de-identified and accessed through the institutional clinical databases. All MRI scans were performed with patients in the supine position. As damage to the SC during epidural catheterization was our main concern, we focused on the spine levels where the SC is theoretically at the highest risk of direct needle trauma. Three investigators from the Canadian center (N.L., D.A., A.H.), as well as three investigators from each of the Brazilian centers (V.C.Q., F.B.C., R.V.C. and L.M.S., F.N B., S.Q.S.) independently collected the following four measurements/distances at levels T5/T6, T9/T10, and L1/L2 (Fig. 1) in a standardized fashion: from the ventral edge of the LF to the dorsal edge of the SC both (1) Perpendicular to the SC as well as (2) At an angle parallel to the inferior spinous process; and from the ventral edge of the dura to the dorsal edge of the SC both (3) Perpendicular to the SC as well as (4) At an angle parallel to the inferior spinous process. All investigators participating in data collection were blinded to each other’s measurements. Notably, measurements at an angle parallel to the inferior spinous process aimed to mimic the epidural needle trajectory. In equivocal scans where the conus medullaris was located in close proximity to L1/L2, all 3 investigators met to review the images and ultimately reach a consensus as to whether the conus medullaris could be reached by a needle traversing the L1/L2 interspace. The mean measurements collected by the 3 independent investigators at each center were used in data analysis, and the Intraclass Correlation Coefficient (ICC) was calculated to assess Inter-Rater Reliability (IRR). Normality of the distribution of each measurement was assessed using the Shapiro-Wilk test and visually inspected using histograms. Given the non-normal distribution observed across the analyzed variables, descriptive statistics are presented as medians and ranges. ICC estimates and their 95% Confident Intervals (95% CIs) were calculated using SPSS statistical package version 23 (SPSS Inc, Chicago, IL, USA) based on a mean-rating (*k* = 3), absolute-agreement, 2-way mixed-effects model. Notably, ICC values < 0.5, between 0.5-0.75, between 0.75‒0.9, and > 0.90 are indicative of poor, moderate, good, and excellent reliability, respectively.^12^ The MRI resolution is approximately 0.1 mm. For production of graphs, Microsoft Excel Version 2016 (Microsoft Inc, Redmond, WA, USA) was used. This was a retrospective descriptive imaging study which adhered to the Strengthening the Reporting of Observational Studies in Epidemiology (STROBE) guidelines.

## Results

The demographic characteristics associated with the MRI scans reviewed are shown in Table 1. All scans were within normal limits as reported by certified radiologists. In total, there were 52 females, and the cohort’s median age was 7 years old. ICC was estimated at 0.929 (95% CI 0.916‒0.940) for the raters at the Canadian center, and 0.920 (95% CI 0.898‒0.939) at the Brazilian centers, indicating excellent IRR.^12^ Male and female data were analyzed together as previous data have shown that there were no significant differences between sexes in our measurements of interest.^9^

**Table 1:** Demographic characteristics of pediatric patients whose MRI images were retrospectively reviewed. Data presented as n (%) or median (IQR).

	Age (years)							
	All ages (n = 111, 100%)	< 1 (n = 8, 7%)	1‒2 (n = 13, 12%)	3‒4 (n = 21, 19%)	5‒6 (n = 13, 12%)	7‒8 (n = 16, 14%)	9‒10 (n = 21, 19%)	11‒12 (n = 19, 17%)
**Median (IQR) age (years)**	7 (3‒10)	0.2 (0.15‒0.44)	1.9 (1.6‒2)	4 (3‒4)	6 (5‒6)	7 (7‒8)	10 (9‒10)	11 (11‒12)
**Male**	59 (53%)	4 (50%)	5 (38%)	9 (43%)	9 (69%)	7 (44%)	12 (57%)	13 (68%)
**Female**	52 (47%)	4 (50%)	8 (62%)	12 (57%)	4 (31%)	9 (56%)	9 (43%)	6 (32%)
**Conus Medullaris above L1/L2**	47 (42%)	4 (50%)	5 (38%)	12 (57%)	2 (15%)	4 (25%)	10 (48%)	10 (53%)
**Conus Medullaris at or below L1/L2**	64 (58%)	4 (50%)	8 (62%)	9 (43%)	11 (85%)	12 (75%)	11 (52%)	9 (47%)

L1/L2, Lumbar 1/Lumbar 2; MRI, Magnetic Resonance Imaging.

The conus medullaris was superior to the L1/L2 vertebral interspace in 47 patients, requiring exclusion of their L1/L2 measurements. There were nine patients in which L1/L2 images were available and included in measurements; however, images were not accessible for T5/T6 or T9/10, and there was one patient with all but T5/6 measurements available. Given the limited nature of data on our topic of investigation, we attempted to include all measurements. When data were not available at all vertebral levels for participants, the remaining data were included with no imputations for missing values. The age distribution among these 111 children is shown in Table 1. Investigators met to review nine equivocal scans where the conus medullaris was in close proximity to L1/L2. Of these, three scans were in the < 1 year-old cohort (of which two were excluded from the L1/L2 measurements), two scans in the 3‒4 year-old cohort (one excluded), one scan in the 5‒6 year-old cohort (not excluded), one scan in the 7‒8 year-old cohort (excluded), and two scans in the 11‒12 year-old cohort (both excluded).

**Figure 2:**
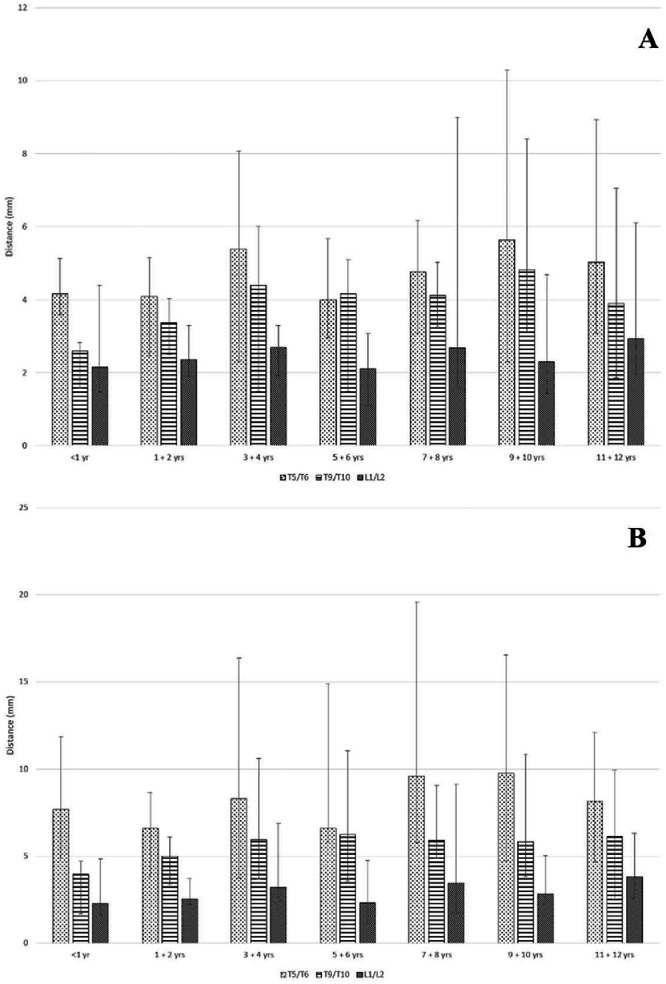
Distances (Median, Range) from the ventral dura mater to the dorsal spinal cord measured perpendicular to the spinal cord (A) and parallel to the inferior spinous process (B) at the T5/T6, T9/T10, and L1/L2 interspaces across age groups.

**Figure 3:**
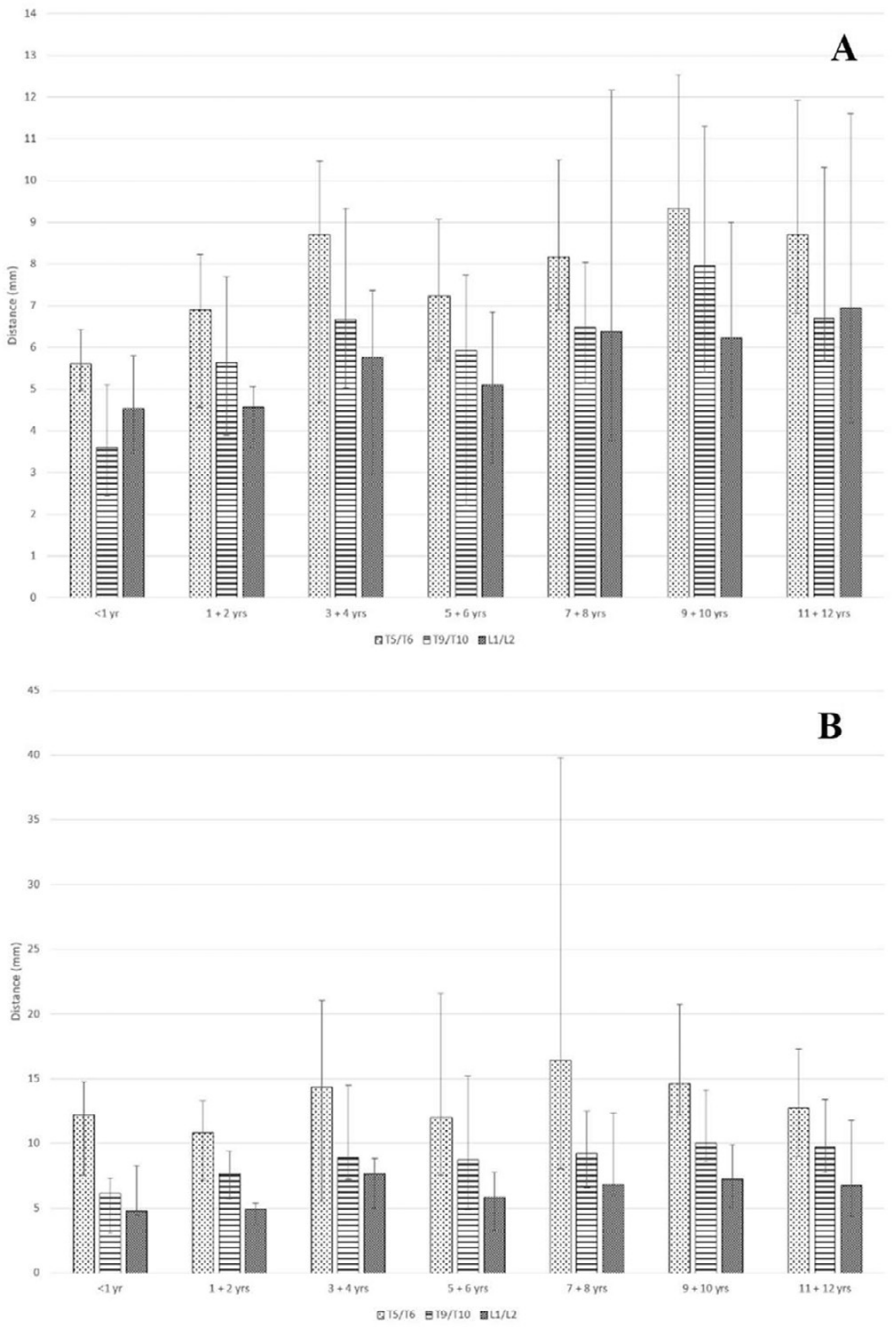
Distances (Median, Range) from the ventral ligamentum flavum to the dorsal spinal cord measured perpendicular to the spinal cord (A) and parallel to the inferior spinous process (B) at the T5/T6, T9/T10, and L1/L2 interspaces across age groups.

Table 2, Table 3 and Figure 2, Figure 3 present our distance results (please note the different y-axis scales in the figures). When measured perpendicular to the SC, the greatest distances (i.e., dura-SC distance and LF-SC distance) for our entire cohort were at T5/T6, as seen in Figures 2A and 3A. Specifically, the median dura-SC distance (measured perpendicular to the SC) in our cohort was 4.87 mm at T5/T6, compared to 4.03 mm at T9/T10 and 2.31 mm at L1/L2; whereas, the median LF-SC distance was 8.10 mm at T5/T6, compared to 6.65 mm at T9/T10 and 5.82 mm at L1/L2.

**Table 2:** Distances (median, range) (mm) from the ventral dura-mater to the dorsal spinal cord measured perpendicular to the spinal cord (A) and parallel to the inferior spinous process (B) at the T5/T6, T9/T10, and L1/L2 interspaces across age groups.

A								
Vertebral Interspace	Age (years)							
	All ages	< 1	1‒2	3‒4	5‒6	7‒8	9‒10	11‒12
T5/T6	4.87 (2.30‒10.30)	4.16 (3.60‒5.13)	4.10 (2.46‒5.16)	5.40 (2.30‒8.07)	4.01 (2.96‒5.67)	4.76 (3.10‒6.17)	5.63 (2.30‒10.30)	5.03 (3.10‒8.92)
T9/T10	4.03 (1.50‒8.40)	2.60 (1.58‒2.83)	3.37 (2.50‒4.03)	4.40 (1.50‒6.00)	4.16 (1.51‒5.10)	4.12 (3.27‒5.03)	4.83 (3.13‒8.40)	3.90 (1.86‒7.05)
L1/L2	2.31 (1.10‒9.00)	2.15 (1.48‒4.40)	2.36 (1.90‒3.30)	2.70 (1.93‒3.30)	2.11 (1.10‒3.07)	2.68 (1.60‒9.00)	2.30 (1.43‒4.70)	2.93 (1.93‒6.10)

B								
Vertebral Interspace	Age (years)							
	All ages	< 1	1‒2	3‒4	5‒6	7‒8	9‒10	11‒12
T5/T6	8.20 (3.75‒19.57)	7.68 (4.87‒11.83)	6.60 (3.80‒8.67)	8.30 (3.75‒16.36)	6.60 (5.80‒14.87)	9.62 (5.78‒19.57)	9.76 (4.73‒16.53)	8.15 (4.66‒12.07)
T9/T10	5.86 (1.69‒11.07)	3.95 (1.69‒4.70)	5.00 (3.37‒6.10)	5.96 (3.70‒10.63)	6.24 (3.57‒11.07)	5.91 (4.87‒9.07)	5.83 (3.83‒10.86)	6.13 (2.53‒9.93)
L1/L2	2.80 (1.16‒9.13)	2.28 (1.61‒4.85)	2.53 (2.23‒3.73)	3.20 (2.43‒6.90)	2.33 (1.16‒4.76)	3.47 (1.77‒9.13)	2.83 (2.23‒5.03)	3.81 (2.60‒6.30)

**Table 3:** Distances (median, range) (mm) from the ventral ligamentum flavum to the dorsal spinal cord measured perpendicular to the spinal cord (A) and parallel to the inferior spinous process (B) at the T5/T6, T9/T10, and L1/L2 interspaces across age groups.

A								
Vertebral Interspace	Age (years)							
	All ages	< 1	1‒2	3‒4	5‒6	7‒8	9‒10	11‒12
T5/T6	8.10 (4.57‒12.53)	5.60 (4.97‒6.43)	6.90 (4.57‒8.23)	8.70 (4.70‒10.46)	7.23 (5.69‒9.06)	8.17 (6.89‒10.50)	9.33 (5.90‒12.53)	8.70 (6.81‒11.93)
T9/T10	6.65 (2.20‒11.30)	3.60 (2.43‒5.10)	5.63 (3.90‒7.70)	6.67 (5.03‒9.33)	5.94 (2.20‒7.73)	6.48 (5.13‒8.03)	7.96 (5.40‒11.30)	6.70 (5.66‒10.32)
L1/L2	5.82 (2.96‒12.16)	4.53 (3.47‒5.80)	4.56 (3.60‒5.06)	5.76 (2.96‒7.36)	5.10 (3.22‒6.85)	6.38 (3.77‒12.16)	6.23 (4.33‒9.00)	6.94 (4.20‒11.60)

B								
Vertebral Interspace	Age (years)							
	All ages	< 1	1‒2	3‒4	5‒6	7‒8	9‒10	11‒12
T5/T6	13.40 (5.50‒39.77)	12.22 (7.53‒14.80)	10.80 (7.16‒13.30)	14.33 (5.50‒21.03)	12.00 (7.57‒21.57)	16.42 (8.06‒39.77)	14.63 (12.16‒20.70)	12.76 (9.02‒17.30)
T9/T10	9.00 (3.03‒15.20)	6.13 (3.03‒7.33)	7.63 (5.67‒9.43)	8.90 (7.23‒14.53)	8.73 (4.87‒15.20)	9.21 (6.57‒12.47)	10.00 (8.60‒14.13)	9.70 (7.79‒13.38)
L1/L2	6.56 (3.27‒12.36)	4.78 (4.43‒8.30)	4.90 (3.76‒5.43)	7.63 (5.00‒8.83)	5.83 (3.27‒7.76)	6.82 (5.99‒12.36)	7.23 (5.07‒9.90)	6.77 (4.33‒11.80)

As expected, the distances were greater when measured at the angle parallel to the inferior spinous process. When considering these particular measurements, the greatest distances (i.e., dura-SC distance and LF-SC distance) in all the age groups were also at the T5/T6 interspace as seen in Figures 2B and 3B. Specifically, the median dura-SC distance (measured at the angle parallel to the inferior spinous process) in our entire cohort was 8.20 mm at T5/T6, compared to 5.86 mm at T9/T10 and 2.80 mm at L1/L2, whereas the median LF-SC distance was 13.40 mm at T5/T6, compared to 9.00 mm at T9/T10 and 6.56 mm at L1/L2.

While the various distances are expected to increase with age, once the subjects reach the ages 3‒4 years, the measured differences from there on to ages 11‒12 years were small and not necessarily linear. This is especially true of the ventral dura to dorsal SC distance, which remains within a small range even as children grow.

## Discussion

The greatest depth of the SC at all ages was at T5/T6, regardless of whether it was measured perpendicular to the SC or parallel to the spinous process,^5^, ^6^, ^7^^,^^9^ which is clinically more relevant. The difference between the perpendicular and parallel distances is greatest at T5/T6 because of the steep angle of the spinous processes. Our results confirm previously reported findings in pediatric patients both at thoracic and lumbar levels.^5^^,^^9^^,^^10^

The mid-thoracic region is at the apex of the thoracic spine curvature, where the SC is located most ventral due to tethering of the dentate ligaments, creating the largest space ventral to the dura and dorsal to the SC at this level.^6^ The epidural space is also largest in the mid-thoracic region. In contrast, the SC at the lower thoracic and lumbar levels are more dorsal because of normal lumbar SC enlargement which generally starts at T11 and ends at L2.^9^ Taken together, this means that the greatest safety margin for epidural needle/catheter placement is in the T5/T6 region. However, this advantage needs to be balanced against the potential damage at a higher SC level, should one occur. Furthermore, the pediatric rib cage being more compliant at the mid-thoracic level may increase the chance of accidentally inserting the needle too far during a recoil.

All distance measurements presented with wide ranges (Table 2, Table 3 and Figure 2, Figure 3). Indeed, as hypothesized (based on adult studies), wide distance ranges were observed, especially at the T5/T6 levels. This is an inconvenient finding that practitioners must bear in mind when performing an epidural, namely that mean distances should not be relied upon to expect when important anatomic structures are reached. Rather, a relatively unpredictable wide distance range is to be expected.

The largest mean differences in the various measured distances between the youngest and the oldest in our cohort are only a mere ∼2‒4 mm, depending on the spinal level and the needle trajectory, in agreement with a previous study that measured dura to SC in children,^9^ suggesting that older children are not significantly less susceptible to inadvertent injury to the SC than very young children.

Our study is the first of its kind to measure the dura-SC and the LF-SC distances in children. Our results complement those in children that measured the skin-dura^5^^,^^11^ and dura-SC^10^ distances. Awareness of the approximate distances between these important structures and their high variability may improve safety.

There is always a risk of inadvertent dural puncture (and ultimately SC injury depending on how far the needle is advanced) during epidural access regardless of the thoracic level. The Pediatric Regional Anesthetic Network reported two dural punctures out of 103 lumbar epidurals and one dural puncture out of 13 thoracic epidurals.^13^ Giaufre et al. recorded two dural punctures in a total of 2,396 pediatric lumbar epidurals.^14^ In a follow-up study, four dural taps were recorded in 1,547 epidurals (ages from 1-month to 13 years), three at the lumbar level (with one catheterization of the subarachnoid space), and one at the thoracic level.^1^ An audit from the U.K. (10,633 pediatric epidurals) reported permanent residual neurologic deficits in a 3-month-old child (at 1-year follow-up) and one post-dural puncture headache.^3^ Direct SC needle trauma^15^, ^16^, ^17^ and inadvertent catheterization of the subarachnoid space^18^ resulting in permanent neurologic deficits have also been reported. Knowing the dura and SC depths may be particularly helpful for novices, who have less experience with tactile sensation. Accordingly, SC injury has been reported in a pediatric patient after a practitioner with 3 years of clinical experience attempted epidural placement. ^15^

With Loss of Resistance (LOR), the epidural needle can be advanced continuously or incrementally. If while utilizing intermittent LOR, the incremental distance is larger than the distance between the ventral aspect of the LF and the dura, the first LOR encountered may be after the dura has been breached (if the needle tip during the resistance check prior to the dural puncture had been just before emerging from the LF). Likewise, if the incremental distance is larger than the LF-SC distance, a needle that had been on the verge of emerging from the LF may reach the SC with the next increment. As such, there may not be the LOR expected in the epidural space or the CSF feedback when dural puncture or SC contact occurs. Although theoretical, this may encourage clinicians to consider using the continuous rather than intermittent LOR technique or reduce their incremental advancing distance in pediatric patients.

The length of the needle bevel opening also deserves consideration. At the study centers, it measured approximately 1.2 millimeters in pediatric Tuohy needles, which may result in an increased safety profile, particularly for the smaller patients. If any portion of this opening is in the epidural space, the operator would expectedly encounter LOR and not advance the needle further.

One limitation of our study is the small sample size due to the low number of apparently normal pediatric spinal MRI scans taken at our centers. This may be particularly important at the L1/L2 interspace, where measurements were performed in 63/111 scans, due to the conus medullaris ending superior to L1 in the remaining ones. Additionally, the studied population (i.e., patients undergoing MRI scan for suspected spine pathology) may not be fully representative of the general pediatric population. Another limitation is patient positioning. All MRI scans were performed with patients in the supine rather than in the lateral position or, less commonly, sitting position, all used for neuraxial blocks. The supine position causes the SC to migrate dorsally with gravity^6^ and may cause compression of subcutaneous tissues, suggesting that the distances measured in our study, including the safety margins, may actually underestimate the distances an operator encounters (and those previously reported)^5^, ^6^, ^7^, ^8^^,^^10^^,^^11^ once the patient assumes an ideal epidural placement position. Furthermore, during neuraxial block, the spine is arched to maximize the inter-spinous process space whereas, during MRI, the spine is not. In adults, there are indeed significant differences in the dura-SC distances between the sitting (slightly hunched back), supine, and lateral recumbent (relatively neutral and not decubitus) positions.^9^ We did not collect data on MRI machine specifications (specifically, resolutions) over the studied period; there was likely lack of standardization of MRI machines among participating centers, thereby resulting in image quality/resolution variation. Lastly, because of the wide distance ranges found and the intrinsic resolution of MRI images, our study does not provide the answer to whether LOR should be sought with incremental vs. continuous advancement of the Tuohy needle in children. In simulation, overshoot of the Tuohy needle is worse (thus theoretically threatening the SC) with the incremental approach.^19^ If an incremental approach is the preferred technique, small increments must be used with each check of LOR.

## Conclusion

Our findings suggest that the margin of safety (i.e., dura-SC distance and LF-SC distance) for performing epidurals in children may be greatest at the mid-thoracic spinal region, regardless of whether it was measured perpendicular to the SC or parallel to the spinous process. Additionally, mean distances should not be relied upon while performing an epidural, given the observed wide distance ranges. Further studies are warranted to validate these findings in pediatric patients with other relevant “epidural placement” positions.

## Authors’ contributions

All authors conceived, drafted and critically revised the manuscript, and approved the final version submitted for publication in the Brazilian Journal of Anesthesiology.

## Data availability statement

The datasets generated and/or analyzed during the current study are available from the corresponding author upon reasonable request.

## Conflicts of interest

The authors declare no conflicts of interest.
